# Postnatal Role of the Cytoskeleton in Adult Epileptogenesis

**DOI:** 10.1093/texcom/tgaa024

**Published:** 2020-06-17

**Authors:** Cezar Gavrilovici, Yulan Jiang, Ivana Kiroski, G Campbell Teskey, Jong M Rho, Minh Dang Nguyen

**Affiliations:** Departments of Neurosciences & Pediatrics, University of California San Diego, Rady Children’s Hospital San Diego, San Diego, CA 92123, USA; Departments of Clinical Neurosciences, Cell Biology & Anatomy, and Biochemistry & Molecular Biology, Hotchkiss Brain Institute, Alberta Children Hospital Research Institute, University of Calgary, Calgary T2N 4N1, Canada; Departments of Clinical Neurosciences, Cell Biology & Anatomy, and Biochemistry & Molecular Biology, Hotchkiss Brain Institute, Alberta Children Hospital Research Institute, University of Calgary, Calgary T2N 4N1, Canada; Department of Cell Biology & Anatomy, Hotchkiss Brain Institute, Alberta Children Hospital Research Institute, University of Calgary, Calgary T2N 4N1, Canada; Departments of Neurosciences & Pediatrics, University of California San Diego, Rady Children’s Hospital San Diego, San Diego, CA 92123, USA; Departments of Clinical Neurosciences, Cell Biology & Anatomy, and Biochemistry & Molecular Biology, Hotchkiss Brain Institute, Alberta Children Hospital Research Institute, University of Calgary, Calgary T2N 4N1, Canada

**Keywords:** cytoskeleton, epileptogenesis, Ndel1, neuronal excitability, seizures

## Abstract

Mutations in cytoskeletal proteins can cause early infantile and childhood epilepsies by misplacing newly born neurons and altering neuronal connectivity. In the adult epileptic brain, cytoskeletal disruption is often viewed as being secondary to aberrant neuronal activity and/or death, and hence simply represents an epiphenomenon. Here, we review the emerging evidence collected in animal models and human studies implicating the cytoskeleton as a potential causative factor in adult epileptogenesis. Based on the emerging evidence, we propose that cytoskeletal disruption may be an important pathogenic mechanism in the mature epileptic brain.

## Introduction

In this review, epilepsy relates to a chronic condition whose hallmark feature is the occurrence of spontaneous recurrent seizures (SRS) (Sperk et al. 2009; Jefferys 2010; Kobow et al. 2012; Swann and Rho 2014) and is typically associated with comorbid conditions, including learning and memory deficits (Bell et al. 2011), anxiety (Tellez-Zenteno et al. 2007), and depression (Ettinger et al. 2004; Fuller-Thomson and Brennenstuhl 2009). With more than 50 million individuals (adults and children) having epilepsy worldwide and with an incidence rate of ~61.4 per 100 000 person-years, epilepsy is one of the most common neurological disorders (Fiest et al. 2017). Epileptogenesis refers to the process by which a typical brain becomes epileptic and self-generates recurrent seizure activity (Dudek and Staley 2012). The term epileptogenesis has also been applied to the process by which epileptiform activity and seizure behaviors become more severe with repetition, a phenomenon also called kindling (Goddard et al. 1969). Kindling is a form of sensitization observed in plastic systems, whereby repeated elicitation of events result in the progressive amplification of a response regardless of whether the events are induced exogenously and are known (electrical, chemical, or optogenetic) or induced endogenously and are unknown (Bertram and Cornett 1993; Wolff et al. 2020). Some of the underlying mechanisms, like those that result in the lowering of seizure thresholds, are likely to be common to both processes (Teskey 2020).

Unfortunately, the mechanisms underlying adult epileptogenesis continue to remain elusive but some of the molecular and cellular processes have emerged from human studies, genetic rodent models of epilepsy and animal models induced by status epilepticus (Guerrini and Carrozzo 2001; Ross et al. 2001; Paredes and Baraban 2002; Scantlebury et al. 2007; Wynshaw-Boris 2007; Rakhade and Jensen 2009; Staley 2015; Khaspekov and Frumkina 2017). Factors such as altered neuronal migration, disturbances of ion channels, excitotoxic cell death, inflammation, astrogliosis, and alterations in dendritic plasticity causing imbalance in inhibitory/excitatory inputs have been proposed as relevant, associated, and/or causative mechanisms for epileptogenesis (Guerrini and Carrozzo 2001; Ross et al. 2001; Paredes and Baraban 2002; LoTurco and Bai 2006; Scantlebury et al. 2007; Wynshaw-Boris 2007; Rakhade and Jensen 2009; Gardiner and Marc 2010; Pramparo et al. 2011; Sutherland-Smith 2011; Stouffer et al. 2016; Wei et al. 2017; Guarnieri et al. 2018; Rana and Musto 2018; Wade et al. 2020). When viewed from the perspective of basic cellular biology, all these changes are directly or indirectly linked to alterations in the cytoskeleton.

The cytoskeleton is the physical backbone that provides structural integrity and functionality to the cell and confers cellular plasticity during periods of adaptation and adversity (Fletcher and Mullins 2010). This structural entity is formed by the interconnection of 3 dynamic intracellular filamentous networks of proteins, that is, microtubules (MTs), intermediate filament (IF), microfilaments (MFs, also called actin filaments), as well as of their associated proteins (termed MT-, IF-, or MF-associated proteins) and molecular motors (dynein and kinesins) (Sharp et al. 2000; Fletcher and Mullins 2010; Xiao et al. 2016; Goodson and Jonasson 2018). Unequivocal evidence for a prenatal role of the cytoskeleton in the pathogenesis of epilepsy has long been established in the pediatric epilepsy population at the genetic level, and is supported by functional assays in animal models with brain malformations (Guerrini and Carrozzo 2001; Ross et al. 2001; Paredes and Baraban 2002; Shu et al. 2004; Sasaki et al. 2005; Toyo-Oka et al. 2005; LoTurco and Bai 2006; Wynshaw-Boris 2007; Youn et al. 2009; Hippenmeyer et al. 2010; Pramparo et al. 2011; Sutherland-Smith 2011; Stouffer et al. 2016; Guarnieri et al. 2018; Wade et al. 2020). In contrast, the postnatal contribution of the cytoskeleton in adult epilepsy remains unclear and controversial. Indeed, cytoskeletal disruption in the adult epileptic brain is thought to result from aberrant neuronal activity and/or death, thereby merely representing an epiphenomenon. The current review summarizes the recent evidence in animal models and human studies suggesting that the cytoskeleton can play a causal role in epileptogenesis in the postnatal brain. While this notion remains to be fully validated, the evidence to date provides novel insights into the pathogenic mechanisms underlying adult epileptogenesis.

## Brain Malformation and Epileptogenesis

A prenatal role of the cytoskeleton in the pathogenesis of epilepsy is well established in the pediatric epilepsy population. Many infants and children with severe, medically intractable epileptic syndromes exhibit prenatal defects in cytoskeletal structure/function that can cause various brain malformations and neuronal migration disorders. Neuronal migration disorders refer to a family of neurodevelopmental diseases characterized by an altered lamination of the brain (Guerrini and Carrozzo 2001; Ross et al. 2001; Paredes and Baraban 2002; Wynshaw-Boris 2007; Sutherland-Smith 2011; Stouffer et al. 2016; Guarnieri et al. 2018; Wade et al. 2020). These include lissencephaly (smooth brain) and cortical dysplasia (abnormal growth or development) caused by mutations in *TUBA1A and TUBG1* (Khodiyar et al. 2007; Poirier et al. 2007; Poirier et al. 2010; Fallet-Bianco et al. 2014; Romaniello et al. 2019) that encode for the alpha, and gamma Tubulin subunit proteins forming the MTs, respectively. Mutations in *TUBB2B, TUBB3* that encode for the beta Tubulin subunit of MTs, give rise to polymicrogyria, a human cortical malformation associated with epilepsy affecting mostly neuronal progenitors in the cortical epithelium (Stottmann et al. 2013; Cushion et al. 2014; Romaniello et al. 2019). Together, these diseases are also referred to as tubulinopathies (Guarnieri et al. 2018; Romaniello et al. 2019). Mutations in Lis1 MT-associated proteins (MAP) are causative of lissencephaly (Reiner et al. 1993; Guerrini and Carrozzo 2001; Leventer et al. 2001; Ross et al. 2001; Paredes and Baraban 2002; Wynshaw-Boris 2007; Saillour et al. 2009) while those in the MAP doublecortin DCX account for the double cortex syndrome (Gleeson et al. 1998; Guerrini and Carrozzo 2001; Ross et al. 2001; Paredes and Baraban 2002; Guerrini and Filippi 2005; Moores et al. 2006; Wynshaw-Boris 2007; Bahi-Buisson et al. 2013; Fu et al. 2013). Finally, genetic variations in the MF-associated protein filamin A are responsible for the classical X-linked form of bilateral periventricular nodular heterotopia (PNH) (Fox et al. 1998; Sutherland-Smith 2011; Lange et al. 2015; Wade et al. 2020). Table 1 summarizes the main genes associated with these brain malformations and neuronal migration disorders, and their links to epilepsy.

**Table 1:** Genes associated with brain malformations and neuronal migration disorders and their link to epilepsy

Mutated gene	Protein	Cytoskeletal function	Phenotypes and clinical symptoms	Animal models	References
*TUBA1A*	Tubulin α-1A	Forms MTs by heterodimerizing with β-tubulin	Lissencephaly; epilepsy, motor delay	Perinatal death and forebrain malformations (enlarged ventricles with hypoplastic basal ganglia and disruption to the ventricular zone, intermediate zone and cortical plate) in KO	(Khodiyar et al. 2007; Poirier et al. 2007; Guarnieri et al. 2018; Bittermann et al. 2019; Romaniello et al. 2019)
*TUBB2B*	Tubulin β-2B	Forms MTs by heterodimerizing with α-tubulin	Brain dysgenesis with bilateral, asymmetrical polymicrogyria	Embryonic lethality and abnormal cortical development (dysmorphic brain with reduced neuroepithelium and ventriculomegaly) in brain dimple (brdp) mice carrying a missense mutation in TUBB2B	(Jaglin et al. 2009; Stottmann et al. 2013; Fallet-Bianco et al. 2014; Guarnieri et al. 2018; Romaniello et al. 2019)
*TUBB3*	Tubulin β-3	Forms MTs by heterodimerizing with α-tubulin	Polymicrogyria-like cortical dysplasia with dysmorphic basal ganglia, brainstem and cerebellar vermian hypoplasia	Decreased growth cone MT dynamics in dorsal root ganglia, delayed peripheral nerve regeneration but no major neuroanatomical or behavioral defects in KO	(Poirier et al. 2010; Cushion et al. 2013; Guarnieri et al. 2018; Latremoliere et al. 2018; Romaniello et al. 2019)
*ACTB*	Actin β	Forms actin filaments	Brain malformation and lissencephaly; seizures, intellectual impairment	Embryonic lethality caused by impaired erythropoiesis leading to hypoxia in KO	(Tondeleir et al. 2013; Di Donato et al. 2014; Verloes et al. 2015)
*ACTG1*	Actin γ-1	Forms actin filaments	Brain malformation and lissencephaly; hearing loss, seizures, intellectual impairment	Reduced body weight and progressive hearing loss characterized by stereocilia degradation in KO	(Belyantseva et al. 2009; Verloes et al. 2015; Kemerley et al. 2017)
*Lis1*	Lissencephaly 1	Regulates the molecular motor cytoplasmic Dynein and MTs organization	Lissencephaly, subcortical band heterotopia (SBH); developmental delay, intellectual disability, epilepsy	Embryonic lethality in KO; neuronal migration defects and seldom seizures in heterozygous KO	(Reiner et al. 1993; Hirotsune et al. 1998; Guerrini and Carrozzo 2001; Leventer et al. 2001; Ross et al. 2001; Paredes and Baraban 2002; Jones and Baraban 2007; Wynshaw-Boris 2007; Greenwood et al. 2009; Saillour et al. 2009)
*DCX*	Doublecortin	Stabilizes MTs and acts as an anticatastrophe MT factor, bundles MTs and regulates actin structure	SBH in female patients and lissencephaly in male patients; epilepsy, cognitive and language impairment, psychomotor delay	Hyperactivity and seizures in KO	(Gleeson et al. 1998; Guerrini and Carrozzo 2001; Ross et al. 2001; Paredes and Baraban 2002; Guerrini and Filippi 2005; Moores et al. 2006; Wynshaw-Boris 2007; Nosten-Bertrand et al. 2008; Bahi-Buisson et al. 2013; Fu et al. 2013)
*EML1*	Echinoderm microtubule-associated protein-like 1	Regulates MTs assembly and organization	SBH, brain malformations; seizures, developmental delay, visual impairment	Subcortical heterotopia with severe brain anomalies (corpus callosum agenesis and hippocampal defects) in KO	(Kielar et al. 2014; Collins et al. 2019; Oegema et al. 2019)
*FLNA*	Filamin A	Cross-linking and branching of F-actin, increases the flexibility of the actin network	PNH; epilepsy and cardiovascular defects in females and prenatal lethality in males	FlnA-KO mice display embryonic lethality however conditional FlnA KO mice can be used to study neuronal and cardiovascular development.	(Fox et al. 1998; Feng et al. 2006; Sutherland-Smith 2011; Lange et al. 2015; Wade et al. 2020)
*KIF1A*	Kinesin-like protein KIF1A	Regulates MT-based transport of synaptic vesicle precursors	Hereditary spastic paraplegia, encephalopathy; seizures	Death within 24 h postbirth; motor and sensory disturbances, neuronal degeneration, decreased densities of synaptic terminals and reduced numbers of synaptic vesicles	(Yonekawa et al. 1998; Esmaeeli Nieh et al. 2015; Tanaka et al. 2016; Guo et al. 2020)
*KIF2A*	Kinesin heavy chain member 2A	Depolymerizes and organizes MTs	Cortical dysplasia, lissencephaly, brain malformation; epilepsy	Death within 24 h postbirth in KO; weight loss, hyperactivity, and severe epilepsy in tamoxifen-inducible conditional KO	(Poirier et al. 2013; Tian et al. 2016; Cavallin et al. 2017; Homma et al. 2018)
*KIF4A*	Chromosome-associated kinesin KIF4A	Organizes MTs during mitosis and for cytokinesis	Facial dysmorphism; intellectual disability, epilepsy	Altered frequency and amplitude of mIPSCs and mEPSCs in rat primary hippocampal depleted of Kif4a by small interference RNA, leading to impaired balance between excitatory and inhibitory drive	(Willemsen et al. 2014; Tang et al. 2018)
*KIF5C*	Kinesin heavy chain isoform 5C	Regulates MT-based transport of GABA_A_ receptors	Malformations of cortical development, microcephaly; intellectual disability, epilepsy	No overt phenotype in KO, possibly due to redundancy of and compensation by other kinesins	(Kanai et al. 2000; Nakajima et al. 2012; Willemsen et al. 2014; Michels et al. 2017)
*RELN*	Reelin	Signals through ApoER2 and VLDLr to regulate MT and actin dynamics	Lissencephaly with cerebellar hypoplasia; intellectual disability, epilepsy	Reln-KO mice (Reeler mice) exhibit developmental and motor defects and can be used to investigate neuronal migration, lamination and synaptic stabilization mechanisms	(Hong et al. 2000; Chang et al. 2007; Dazzo et al. 2015; Lossi et al. 2019)

MTs, microtubules; ApoER2, Apolipoprotein E receptor 2; VLDLr, very low-density lipoprotein receptor; KO, knockout mice; mIPSCs, miniature inhibitory postsynaptic currents; mEPSCs, miniature excitatory postsynaptic currents.

MAPs regulate the assembly and dynamics of MTs, and coordinate activities of molecular motors such as dyneins and kinesins (Akhmanova and Steinmetz 2015; Bonini et al. 2017; Ramkumar et al. 2017; Goodson and Jonasson 2018). MF-associated proteins regulate the dynamics of actin through polymerization, capping, and severing. In particular, filamin A has the unique ability of holding 2 filaments of actin together, thereby organizing the actin network (Sutherland-Smith 2011; Wade et al. 2020). Generally speaking, these mutated proteins affect the dynamic structure of the cytoskeleton and alter the ability of neuronal progenitors to divide and/or early-born neurons to adapt their morphology during neuronal migration, thereby preventing them from reaching their correct destinations in the developing brain (Guerrini and Carrozzo 2001; Ross et al. 2001; Paredes and Baraban 2002; LoTurco and Bai 2006; Wynshaw-Boris 2007; Sutherland-Smith 2011; Guarnieri et al. 2018; Wade et al. 2020).

Studies in genetic rodent models harboring deficiencies in these MAPs or MF-associated proteins have enhanced our understanding of how prenatal abnormalities in the cytoskeleton lead to epilepsy (Guerrini and Carrozzo 2001; Ross et al. 2001; Paredes and Baraban 2002; Shu et al. 2004; Sasaki et al. 2005; LoTurco and Bai 2006; Wynshaw-Boris 2007; Youn et al. 2009; Sutherland-Smith 2011; Stouffer et al. 2016; Guarnieri et al. 2018; Wade et al. 2020). For example, nuclear translocation, a key process for locomotion—that is, the coupling of the extension of the leading process to the forward movement of the nucleus toward the pial surface, followed by the pulling of the tail—during radial migration is disrupted in *Lis1*-null mice as well in mice depleted of its binding partners Ndel1 MAP (see below) or dynein heavy chain molecular motor (Shu et al. 2004). Neuronal locomotion is also impaired by missense variants of TUBG1 (Ivanova et al. 2019). Furthermore, the transition from multipolar to bipolar morphology in the intermediate zone or subventricular zone is affected by filamin A, Lis1, and DCX mutations (LoTurco and Bai 2006). These migration-deficient neurons end up at the wrong place and establish connections with the wrong partners. These mechanisms have been reviewed thoroughly in numerous publications for their implication in epilepsy (Guerrini and Carrozzo 2001; Ross et al. 2001; Paredes and Baraban 2002; LoTurco and Bai 2006; Wynshaw-Boris 2007; Sutherland-Smith 2011; Stouffer et al. 2016; Guarnieri et al. 2018; Wade et al. 2020). In brief, there is plethora of evidence indicating a prenatal role for the cytoskeleton in brain malformations and the pathogenesis of pediatric epilepsy.

## Emerging Role of the Cytoskeleton in Seizure Activity in the Adult Brain

In the adult epileptic brain, cytoskeletal alterations are generally viewed as epiphenomena, compensatory, or wholly unrelated to epileptogenesis. These changes include reduced levels of tubulin alpha-1 chain, beta-tubulin, profilin II, neuronal tropomodulin, and phosphorylated MAP 2 in mesial temporal lobe epilepsy (TLE), the most common seizure disorder in adults (Yang et al. 2006). Changes in the levels and activity of enzymes regulating the cytoskeleton such as Akt kinase, C-Abl kinase, and Rac1 small GTPase have also been reported in TLE patients but no evidence of pathogenicity has been forthcoming (Chen et al. 2014; Li et al. 2016; Valmiki et al. 2020). Our understanding of the postnatal roles of the cytoskeleton in adult epilepsy has been further hindered by the embryonic lethality or absence of overt phenotypes of numerous knockout (KO) animals for cytoskeletal proteins, the latter possibly due to compensatory changes. Of particular interest, Lis1 KO mice are embryonic lethal and only a subset of heterozygote Lis1 null mice are susceptible to seizures (Hirotsune et al. 1998; Jones and Baraban 2007; Greenwood et al. 2009). Further, mice with LIS1 haploinsufficiency in adulthood do not show an overt brain lamination phenotype (Hunt et al. 2012) but display postdevelopmental axonal transport defects (Hines et al. 2018), further complicating our understanding of the postnatal role of the cytoskeleton in adult epileptogenesis. Our recent studies reveal that several aspects of neuronal integrity, excitability, circuit function, and molecular marks reminiscent of adult epilepsy are apparent in mice with an early postnatal deletion of the Ndel1 gene in the forebrain (Jiang et al. 2016; Kiroski et al. 2020). Importantly, these mice consistently display SRS that can be assessed electro-clinically, and they also robustly exhibit spatial learning and memory deficits (Jiang et al. 2016; Kiroski et al. 2020).

Ndel1 (pronounced “noodle-1”) is a MAP initially characterized in the context of intracellular transport and MT dynamics (Chansard et al. 2011). It is now recognized as an integrator of the cytoskeleton impacting MTs, IFs, and MFs altogether (Chansard et al. 2011). Ndel1 is required for the activation of the Lis1/Dynein motor complex that is critical for MT organization and intracellular transport of cargoes (McKenney et al. 2010; Reddy et al. 2016). Through these mechanisms, Ndel1 regulates nucleokinesis and promotes neuronal migration in the developing neocortex: Ndel1-depleted neocortices by in utero electroporation of siRNA manifest problems in nuclear translocation, thereby resulting in reduced locomotion and ending up misplaced in the cortical plate (Shu et al. 2004). Ndel1 constitutive KO mice are embryonic lethal around E6.5–E8.5 (Sasaki et al. 2005), precluding a clear role for Ndel1 in adult epileptogenesis. Interestingly, pilocarpine-induced status epilepticus in adult rodents deregulates the expression of Ndel1 (Wu et al. 2014; Choi et al. 2016).

We have recently created forebrain excitatory neuron-specific KO mice for Ndel1 (Jiang et al. 2016) by breeding CaMKIIα-Cre transgenic mice line 29-1 with Ndel1-LoxP mice generated previously (Sasaki et al. 2005). In these mice, the Ndel1 gene is excised between P17 and P21; at ~1 month of age, the Ndel1 protein is knocked down. The dorsal CA1 hippocampus of these mice termed “Ndel1 CKO” mice is particularly vulnerable to anatomic deterioration (Jiang et al. 2016). In adulthood, these mutant mice exhibit fragmentation of MT structure in CA1 pyramidal neurons that cause dendritic and synaptic pathologies and render them, hyperexcitable (Jiang et al. 2016; see Table 2 for a summary of the phenotypes). Ndel1-knocked down CA1 pyramidal neurons also undergo postnatal dispersion around P17, independently of neuronal migration defects; this phenotype arises well after the formation of the CA1 and is exacerbated as the Ndel1 CKO mice age (Jiang et al. 2016). The neocortex remains, however, unperturbed in histological examinations. The cellular, molecular, and anatomical abnormalities in the CA1 are likely responsible for the spatial learning and memory deficits observed in the mutant mice (Jiang et al. 2016; Kiroski et al. 2020). Importantly, using video-electroencephalogram (EEG) recordings, we discovered that Ndel1 CKO develop SRS around 6–7 weeks of age and die from these intense SRS (Kiroski et al. 2020). The average lifespan of the mutant mice is ~10 ± 4 weeks. As indicated by the consistent recordings with depth electrodes, the seizure activity originates from the hippocampus (most likely from the bilayered CA1) and can propagate up to the neocortex as detected time-to-time by the surface electrodes (Kiroski et al. 2020). This epileptic phenotype is consistent with the well-known importance of brain layer formation and maintenance that is disrupted postnatally in the Ndel1 CKO hippocampus. Thus, based on findings in this animal model, Ndel1 is implicated in postnatal epilepsy with potential contribution to adult epileptogenesis.

**Table 2:** Summary of Ndel1 CKO phenotypes and beneficial effects of Reelin

Phenotype	Analysis	Experiment at	Analysis at	Effects of Reelin
Microtubule fragmentation in CA1 pyramidal neurons	Electron microscopy	7 weeks	8 weeks	Significant rescue
Dendritic pathology of CA1 pyramidal neurons	Golgi staining and software-based reconstruction	7 weeks	8 weeks	Significant rescue
Synaptic pathology of CA1 excitatory and inhibitory neurons	Electron microscopy	7 weeks	8 weeks	Significant rescue
CA1 intrinsic hyperexcitability and reduced inhibitory drive	Patch-clamp recordings on brain slices	8 weeks	~9 weeks	Partial rescue
Postnatal dispersion of CA1 pyramidal neurons	Immunolabeling with CA1-specific marker Wfs1	7 weeks	8 weeks	Partial rescue (25%)
Spontaneous recurrent seizures	Video-EEG and Racine scale	6–7 weeks	7–8 weeks	Not determined
Spatial learning and memory deficits	Morris water maze task	7 weeks	8 weeks	Significant rescue
Shortened lifespan (10 ± 4 weeks)	Survival curve	7 weeks	Up to 30 weeks	Doubled lifespan

Wfs1, Wolframin.

**Figure 1:**
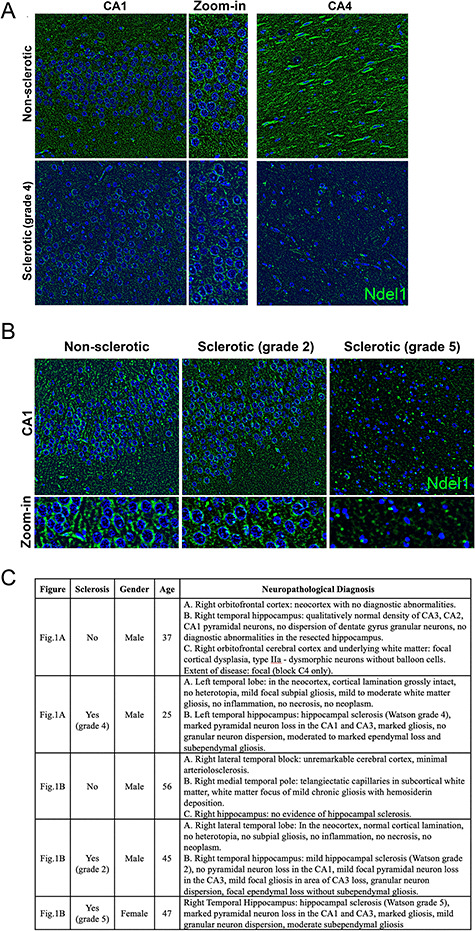
Decreased expression of Ndel1 protein in the human epileptic sclerotic hippocampus. (*A*) Nonsclerotic hippocampus (CA1 and CA4) of a 37-year-old male subject with medically refractory epilepsy versus a Watson grade 4 left sclerotic hippocampus (CA1 and CA4) of a 25-year-old male subject with medically refractory left TLE. The levels of Ndel1 are significantly lower in the latter sample. (*B*) Nonsclerotic hippocampus (CA1) of a 56-year-old male subject with right TLE versus a Watson grade 2 right sclerotic hippocampus (CA1) of a 45-year-old male subject versus a Watson grade 5 right sclerotic hippocampus (CA1) of a 47-year-old female with bilateral-TLE. Nonsclerotic hippocampi show no lesions and no obvious abnormalities. Note the more frequent condensed chromatin in tissues from subjects with severe sclerosis (Watson grades 4 and 5). These surgically resected tissues (sclerotic vs. nonsclerotic) were fixed in formalin and the optimum tissue blocks were chosen by the neuropathologists. Paraffin-embedded hippocampal tissues were prepared by Calgary Laboratory Services. Nonsclerotic patients were matched by age and sex as closely as possible to sclerotic patients. Study protocol and ethics were approved by the Conjoint Health Research Ethics Board at the University of Calgary. A neuropathology report, specifying specimen description, clinical information, diagnosis, gross microscopy and initial pathologist comment, are available upon request for all resected tissues. The identity of the samples was masked until the analysis was completed. Immunolabeling and staining were performed according to published reports (Jiang et al. 2016; Kiroski et al. 2020). (*C*) Summary of patient age, gender, hippocampal sclerosis, and neuropathological diagnosis.

The postnatal dispersion of CA1 pyramidal cells in the Ndel1 CKO mice is reminiscent of granule cells in brains treated with antibodies directed against the glycoprotein Reelin (Heinrich et al. 2006). Mutations in “Reelin” that reduce the protein levels have been reported in familial forms of TLE (Heinrich et al. 2006; Dazzo et al. 2015)—see Table 1. In the developing brain, Reelin regulates neuronal migration via control of cell adhesion and cytoskeletal organization (Sekine et al. 2014; Chai and Frotscher 2016). In the postnatal brain, Reelin is secreted by interneurons and it specifies CA1 pyramidal cell maturation (Kupferman et al. 2014). Reelin also regulates neuronal plasticity (Beffert et al. 2005; Qiu et al. 2006; Qiu and Weeber 2007), increases neurotransmission (Bal et al. 2013), and enhances long-term potentiation in hippocampal slice cultures (Weeber et al. 2002). Interestingly, the levels of Reelin decrease in the Ndel1 CKO hippocampus and its in situ replenishment partially stabilizes the ultrastructural MT fragmentation, alleviates dendritic/synaptic pathology and hyperexcitability of CA1 pyramidal neurons, and reduces their dispersion (Jiang et al. 2016). Further, Reelin ameliorates spatial learning and memory, and even doubles the lifespan of mutant Ndel1 mice (Kiroski et al. 2020; see Table 2 for a summary of the beneficial effects of Reelin in the Ndel1 CKO mice). Since Ndel1 CKO die from SRS and Reelin extends the lifespan of Ndel1 CKO, Reelin may protect against seizure activity. In summary, the Ndel1 CKO mouse strain represents a complex model of epilepsy with multiple underlying molecular and cellular changes. The most direct mechanism that may underlie epileptogenesis in the mutant animals is the intrinsic hyperexcitability of CA1 pyramidal neurons that arises from the progressive dendritic and synaptic pathologies, all consistent with the observed ultrastructural defects in MT structure. This mechanism would align with the findings that Nde1l, the cytoskeleton as well as molecular motors, play a key role in the axon initial segment (AIS), a nevralgic point of control for neuronal polarity, intrinsic neuronal excitability, and a hotspot for epileptogenesis (see section below). Other potential nonmutually exclusive parallel mechanisms of epilepsy include the dispersion of the CA1 that occurs 2 weeks before the first seizures, neuronal death, and inflammation (see sections below). It is noteworthy that the Ndel1 CKO mice affords us not only the exciting opportunity to study the postnatal role of the MT cytoskeleton in epileptogenesis, but also it will enable us to further promote the development of novel therapeutic agents for the treatment of epilepsy, as exemplified by our Reelin experiments.

**Figure 2:**
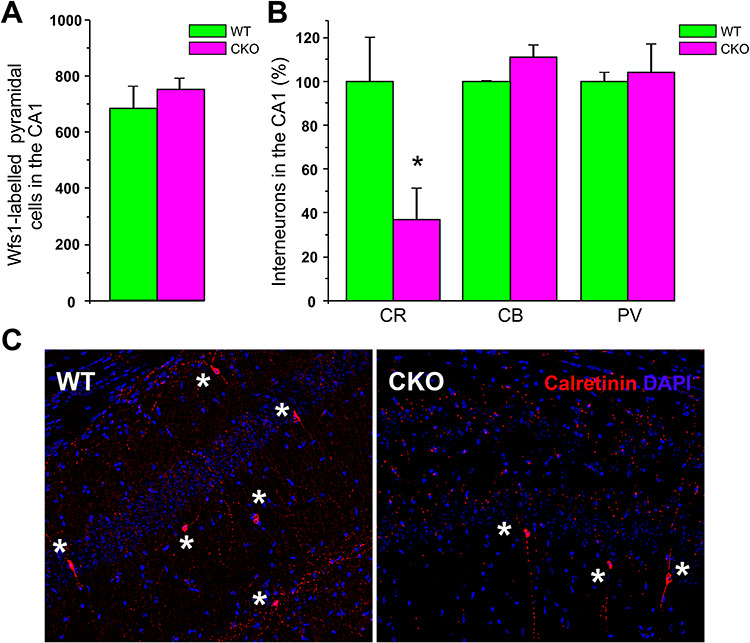
Counts of CA1 principal neurons and interneurons in the Ndel1 CKO hippocampus. (*A*) There is no difference in the number of WFS1-positive CA1 pyramidal neurons in ~12-week-old Ndel1 CKO mice when compared with age-matched wild-type littermates (CKO: 751 ± 42 vs. WT: 682 ± 84; average ± SE, *n* = 3 per genotype, Student’s *t*-test, NS: nonsignificant). Cell counts, labeling, and staining were performed according to (Jiang et al. 2016). (*B*) Selective loss of calretinin (CR)-positive interneurons in ~12-week-old Ndel1 CKO mice when compared with age-matched wild-type littermates. There were no significant differences in the number of calbindin (CB) and parvalbumin (PR)-positive interneurons between the hippocampus of Ndel1 CKO mice and that of wild-type littermates. Calretinin-positive interneurons, CKO: 37 ± 14 versus WT: 100 ± 20; calbindin-positive interneurons, CKO: 111 ± 6 versus WT: 100 ± 1; parvalbumin-positive interneurons, 104 ± 13 versus WT: 100 ± 4; average ± SD, *n* = 4 per genotype, Student’s *t*-test, *P < 0.0; NS: nonsignificant. (*C*) Immunolabeling of CR-positive interneurons in the hippocampus of Ndel1 CKO mice and wild-type littermates. Labeling was performed according to published methods (Jiang et al. 2016; Kiroski et al. 2020).

While the preclinical studies mentioned above provide compelling data, they can only be fully understood and made relevant to the human condition when interpreted in the context of clinical evidence. To gain further insights into the role of Ndel1 in adult epileptogenesis, we analyzed the expression of Ndel1 in surgically resected human epileptic hippocampi with prominent sclerosis (*n* = 4) and compared them with control nonsclerotic CA1 hippocampi from epileptic patients (*n* = 3) (Fig. 1). Ndel1 levels in sclerotic CA1 hippocampi were decreased compared with nonsclerotic specimens. Intriguingly, the extent of this decrease correlates with the severity of the sclerosis. For example, a Watson grade 2 case shows a subtle reduction in Ndel1 levels versus a grade 4 or 5 (most severe) case that exhibits significant downregulation of Ndel1 when compared with their respective controls. As the severity of the sclerosis worsens, the decrease in Ndel1 becomes obvious in the CA4 region, consistent with the higher Watson grades. More frequent condensed chromatin in nuclei observed with DAPI staining was also found in the most severe cases of sclerosis (Fig. 1). The reduced Ndel1 staining in the higher Watson stages (4 and 5) may simply be the result of extensive cell death and gliosis. Alternatively, the loss of Ndel1 in these neurons may predispose them to cell death. This assumption is supported by the findings of degeneration of cortical neurons depleted of Ndel1 by siRNA at E17 and analyzed in the cortical plate at P4 (Nguyen et al. 2004), deterioration of Ndel1 CKO CA1 pyramidal neurons (Jiang et al. 2016; Kiroski et al. 2020) as well as secondary death of calretinin-positive interneurons in the Ndel1 CKO hippocampus (Fig. 2). In this perspective, the decrease of Ndel1 in human epileptic sclerotic hippocampus would be mimicked by the Nde1l CKO hippocampus, further strengthening the scientific rationale for using these mutant mice for modeling human-disease mechanisms.

An important question is whether Ndel1 is the only pertinent cytoskeletal target for adult epilepsy. The answer is definitely not. Our central hypothesis implicating the cytoskeleton as a causative factor in adult epileptogenesis is in line with reports showing that subjects with TLE display MT abnormalities (Yang et al. 2006; Xu et al. 2016). Theses MT changes do not represent epiphenomena, supported by experiments showing that MTs depolymerization exacerbates the severity and prolongs the duration of SRS in 2 rat models of adult epilepsy (i.e., pentylenetetrazol [PTZ]-kindling and pilocarpine) (Xu et al. 2016). Furthermore, a recent report established that 31 out of 33 epileptic patients (94%) exhibited hyperphosphorylation of the MAP Tau (Fyfe 2016; Tai et al. 2016). Hyperphosphorylated Tau detaches from MTs and renders them unstable (Ballatore et al. 2007; Morris et al. 2011). Most importantly, the extent of Tau hyperphosphorylation correlated with cognitive dysfunction in these patients (Fyfe 2016; Tai et al. 2016). Taken together, these studies point to the key role that the cytoskeleton may play in the processes underlying adult epileptogenesis and that the cytoskeleton may be a viable therapeutic target.

### Cytoskeleton-Dependent Mechanisms of Seizures in the Adult Brain

As suggested by functional studies in the adult mouse brain, the cytoskeleton can modify the course and severity of seizure activity and its disruption can even cause adult-onset epilepsy. Altering the cytoskeleton likely triggers seizures through multiple mechanisms related to neuronal dyshomeostasis and neurodegenerative processes (Staley 2015; Holmes and Noebels 2016). In the sections below, we will review and expand upon these mechanisms.

### Neuronal Integrity, Positioning, and Function

The most obvious mechanism underlying the loss of neuronal integrity stems from a structural point of view: A destabilization of the cytoskeleton, and in particular MTs, can collapse the structural architecture of the nerve cell and causes the deterioration of neuronal processes (Jiang et al. 2016; Kiroski et al. 2020). In parallel, axonal transport defects might impede the delivery of essential nutrients necessary for survival and/or signaling molecules required for the preservation of subcellular compartments (Jiang et al. 2016; Hines et al. 2018). Together, these defects can damage excitatory and inhibitory synapses and ultimately promote cellular disconnection and hyperexcitability (Jiang et al. 2016; Hines et al. 2018). Transcriptome analysis of the Nde1l CKO hippocampus (vs. WT vs. Ndel1 CKO hippocampus treated with Reelin) further revealed the importance of genes encoding cell–cell adhesion and contact proteins (such as neurexins and cadherins) (Jiang et al. 2016; Kiroski et al. 2020). While these cellular adhesion molecules are known to contribute to neuronal migration, they now appear to be important for the maintenance of neuronal positioning. Deregulation of these molecules may therefore contribute to the postnatal loss of neuronal positioning in the Ndel1 CKO CA1 and consequently, exacerbate the epileptic phenotype. It is noteworthy subtle mutations in key cytoskeleton and molecular motor genes may not alter neuronal positioning but rather impact fundamental processes such as axonal transport and excitability in an age-dependent manner. Along the same line, as reported for mTOR and PIK3Ca signaling molecules (Koh and Lee 2018), somatic mutations in these genes may occur de novo in subsets of adult neurons giving rise to a mosaic of malfunctioning neurons and therefore, trigger seizures in local brain areas.

### Neuronal Death

Intrinsic neuronal hyperexcitability and glutamate-mediated excitotoxicity can cause imbalance in excitatory/inhibitory inputs in the epileptic brain. When these mechanisms are overwhelming, they induce neuronal death (necrosis, apoptosis, or autophagy) and further accentuate seizure activity (Barker-Haliski and White 2015; Ambrogini et al. 2019; Mao et al. 2019; Hanada 2020). Overactivated N-methyl-D-aspartate (NMDA) receptors by glutamate play a key role in this neurodegenerative process by promoting excess of calcium influx, activating calcium-dependent proteases and generating oxidative stress, while α-amino-3-hydroxy-5-methyl-4-isoxazolepropionic acid (AMPA) receptor hyperactivation participates in the induction of seizures with implication of brain damage (Barker-Haliski and White 2015; Ambrogini et al. 2019; Mao et al. 2019; Hanada 2020). Despite the deterioration of CA1 Ndel1 CKO pyramidal neurons, these cells do not undergo cell death. Indeed, no difference was found in the number of Wfs1-labeled CA1 principal neurons between 12-week-old Ndel1 CKO and wild-type littermates (Fig. 2). Thus, CA1 pyramidal cell death per se does not account for the epileptic phenotype and comorbid memory deficits in Ndel1 CKO mice. However, we surmise that CA1 neuronal death may have become apparent had the Ndel1 CKO mice survived long enough. Interestingly, at the same age, the number of calretinin-positive (but not parvalbumin- or calbindin-positive interneurons) decreased significantly in the hippocampus of Ndel1 CKO mice (Fig. 2). The selective vulnerability to death of calretinin-positive interneurons in the hippocampus of Ndel1 CKO mice is intriguing and could come from the seizure-induced gliosis (see noncell autonomy notion discussed below). Our finding is consistent with studies showing that calretinin-positive neurons are vulnerable in human TLE (Toth and Magloczky 2014). Thus, the combined deterioration of CA1 pyramidal cells, interneuron dysfunction and calretinin-neuron death are likely involved in the enhanced excitability of the hippocampus and seizure activity in the Nde1l CKO mice (Jiang et al. 2016; Kiroski et al. 2020). As pilocarpine treatment can deregulate expression of cytoskeletal proteins (Chen et al. 2014; Wu et al. 2014; Choi et al. 2016) and trigger neuronal death (Xiong et al. 2015; Xiong et al. 2018), this agent may be used to model some aspects of cytoskeletal dysfunction and ultimately, to study cytoskeleton-mediated neuronal death in adult epileptogenesis.

### Inflammation

**Figure 3:**
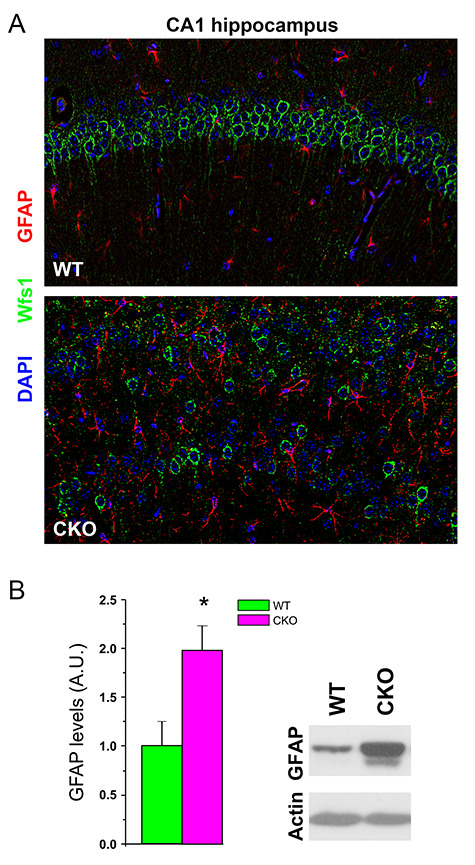
Astrogliosis in the brain of Ndel1 CKO mice. (*A*) Immunolabeling of the IF GFAP (red) Wolframin (Wfs1, green), and nuclei (DAPI, blue) in the hippocampus of Ndel1 CKO mice and wild-type littermates. Note the upregulation and intercalation of GFAP-positive astrocytes between the CA1 bilayer labeled with Wfs1 in the Ndel1 CKO mice. (*B*) Western blots and quantification for the levels of GFAP in the hippocampus of Ndel1 CKO mice (*n* = 4) and wild-type littermates (*n* = 3). *P < 0.05, Student’s *t*-test. Labeling and western blots were performed according to (Nguyen et al. 2004).

Upon parenchymal brain challenge, brain trauma, infection, or blood–brain-barrier disruption, inflammatory molecules released from brain resident cells or peripheral cells can facilitate seizure activity (Vezzani et al. 2011; Galic et al. 2012; Vezzani et al. 2016). For example, the pro-inflammatory cytokines interleukin-1 β (IL-1 β) and tumor necrosis factor-α (TNF-α), secreted by astrocytes and microglia are thought to change neuronal excitability through increased expression of NMDA and AMPA receptors (Viviani et al. 2003; Stellwagen et al. 2005; Galic et al. 2012). Both cytokines can also decrease GABA-mediated neurotransmission (Viviani et al. 2003; Stellwagen et al. 2005; Roseti et al. 2015), thereby unbalancing excitatory/inhibitory drive. In the Ndel1 CKO mice, noncell autonomous mechanisms appear to contribute to epileptogenesis—that is, the mechanisms leading to seizures are not inherent to Ndel1 CKO CA1 pyramidal cell defects per se. This notion is supported by the fact that calretinin-positive interneurons of the Ndel1 CKO mice undergo degeneration even though the Ndel1 gene is not disrupted in these neurons. In this regard, inflammation (i.e., astrogliosis and microgliosis) is likely to play an important role in seizure activity and/or neurodegeneration. Upregulation of the IF glial fibrillary associated protein (GFAP) has been reported in the human epileptic brain and the associated increase in astrogliosis is hypothesized to reflect deleterious inflammation and possibly altered tripartite synapses in epileptic patients (Khaspekov and Frumkina 2017). Consistent with this view, Ndel1 CKO hippocampi display significantly higher levels of GFAP and these reactive astrocytes are found intercalated between the bilayer Ndel1 CKO hippocampus (Fig. 3), a phenotype that is consistent with the altered neuronal connectivity and seizure activity. The proinflammatory mediators secreted by glial cells in the Ndel1 CKO brain and their mechanisms of action (through NMDA, AMPA, and/or GABA receptors for example) remain to be identified. It is noteworthy that the inflammation can also originate from peripheral tissues such as the vascular system that coexists with the cerebral environment to form the blood–brain-barrier, and disruption of the barrier has been shown to contribute to seizure activity (Rana and Musto 2018; Loscher and Friedman 2020). Within the brain, activated resident microglia expressing specific ligands can promote the infiltration of circulating monocytes that contribute to comorbidity after status epilepticus (Varvel et al. 2016). In certain conditions, controlled neuroinflammation is thought to be beneficial for the diseased brain (Kielian 2014). Dissecting these opposing effects of the inflammatory response and its origin will help to clarify the roles of this process in adult epileptogenesis and related comorbidities.

## The Axon Initial Segment: The Missing Link Between Channelopathies and Cytoskeleton Abnormalities?

At the molecular level, ion channels are considered the most relevant targets for epilepsy therapeutic development and for a mechanistic understanding of seizure genesis. Indeed, most antiseizure drugs are designed to modulate ion channels (Meisler et al. 2010; Rajakulendran et al. 2012; Robbins and Tempel 2012; Rogawski et al. 2016). Many patients are managed with these drugs but ~30% remain refractory to pharmacological treatment (Begley et al. 2000), especially TLE patients. Moreover, antiseizures drugs provide only symptomatic relief, and none are preventative or antiepileptogenic. As depolymerization of the MT cytoskeleton can exacerbate the severity and prolongs the duration of SRS in 2 rat models of adult epilepsy (i.e., PTZ kindling and pilocarpine) (Xu et al. 2016), one may consider the use of MT-stabilizing drugs to reduce the severity of epilepsy and associated comorbidities. For example, the brain-penetrant MT-stabilizing drug Epothilone D (EpoD) improves memory deficits in a mouse model of Alzheimer’s disease (Brunden et al. 2010; Brunden et al. 2012; Zhang et al. 2012). MTs-stabilizing agents have also been advanced for the treatment of neurodevelopmental disorders (Bonini et al. 2017) including neuronal migration disorders that are linked to epilepsy. Finally, as channels and receptors are transported on MTs by kinesin and dynein molecular motors, disruption of the cytoskeleton may provide a unifying foundation for the “channelopathy” theory proposed in epilepsy. In support of this view, the transcriptome in the hippocampus of Ndel1 CKO mice revealed that the levels of the voltage-dependent Ca^2+^ channel subunit Cacna2d2, encoded by *CACNA2D2* and mutated in families with epileptic encephalopathy (Edvardson et al. 2013; Pippucci et al. 2013; Butler et al. 2018), are altered (Jiang et al. 2016). Furthermore, mutations in the kinesin family members Kif1a, Kif4a, and Kif5c have been found in patients with epilepsy (Willemsen et al. 2014; Esmaeeli Nieh et al. 2015; Michels et al. 2017). Patient-derived mutant Kif1a increases excitatory synaptic transmission that can contribute to seizure activity (Guo et al. 2020), while mice having a deletion of *KIF5A* exhibit seizures due to altered trafficking of GABA_A_ receptors and impaired GABA_A_ receptor-mediated synaptic transmission (Nakajima et al. 2012). In this regard, MTs binding to GABA_A_ receptor regulates GABA-binding affinity while MT depolarizing agents disrupts GABA_A_ receptor/MT interaction and consequently, inhibit receptor activity (Whatley et al. 1994; Whatley and Harris 1996). Similar functional interactions appear between the actin cytoskeleton and AMPA and NMDA receptors (Hanley 2014; Shaw and Koleske 2020). How the cytoskeleton interacts, localizes, processes, and regulates the functions of these epilepsy-associated channels and receptors in concert with molecular motors to impact adult epilepsy remains to be fully determined. The recent investigations of the molecular network that underlies the AIS has begun to address some of these outstanding questions.

**Figure 4:**
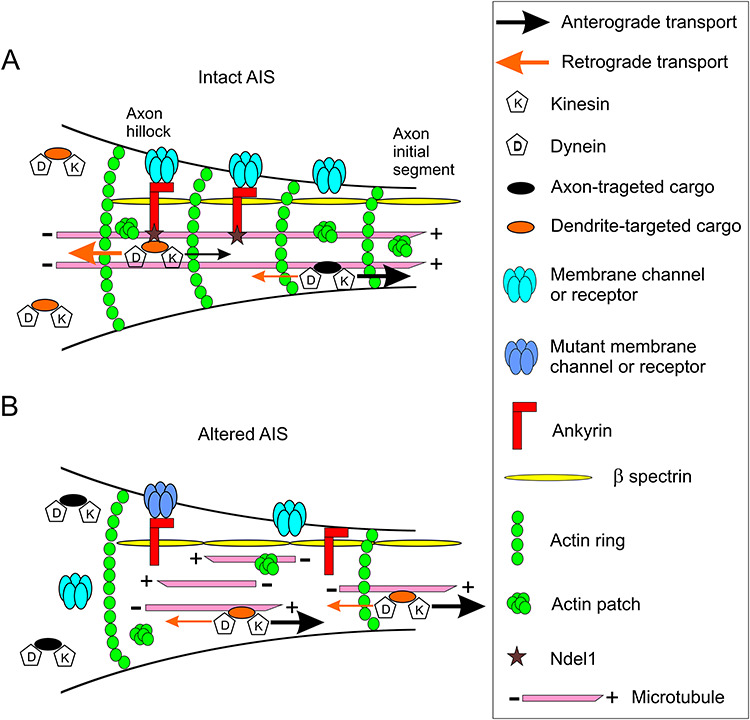
The AIS as a potential missing link between cytoskeletal disruption and channelopathies in adult epileptogenesis. (*A*) Collapse of the cytoskeleton (caused mutations or alterations in cytoskeletal proteins such as Ndel1), and cytoskeleton-dependent aberrant localization of channels and receptors (sodium channel – α1, α2, α6, β1 subunit; K_v_1.1 channel; KCNQ2/3 channel; Ca_V_3.2 channel; GABAA receptor – γ2, α1, β3 subunit; for a review, see Wimmer et al. 2010) can disrupt the integrity and function of the AIS, a hub for the control of neuronal polarity, intrinsic neuronal excitability and potential intracellular site for adult epileptogenesis. (*B*) At the AIS, ankyrin is linked to cytoskeletal actin via β spectrin; the adaptor also binds to Ndel1 that regulates the MT-based dynein-dependent retrograde transport of cargoes, and anchors several membrane channels (e.g., KCNQ2/3, Na_v_, and K_v_7 channels; Nelson and Jenkins 2017; Leterrier 2018) and GABA_A_ receptors (Gao and Heldt 2016). Other channels such Ca_v_ and K_v_1.1 do not require ankyrin for proper membrane localization. (*C*) As Ndel1 regulates the 3 cytoskeletal networks (Chansard et al. 2011), depletion of Ndel1 may lead to cytoskeletal disruption. This includes MT fragmentation (Jiang et al. 2016), abnormal MT polarity, accumulation of neurofilament at the axon hillock (Nguyen et al. 2004), reduced number of actin rings, and mislocalized actin patches that normally block the transport of dendrites-specific cargoes down the axon. Ultimately, these cytoskeletal defects will aberrantly traffic receptors and channels the wrong location. In this context, loss of Ndel1 function also reduces dynein activity that is required to push back the transport of dendrites-specific cargoes down the axon. In a nonexclusive scenario, mutant channels or receptors may also be mislocalized and their functions may also be altered.

The AIS located in the proximal region of the axon right after the axon hillock (the first 20–60 microns of the axon) constitutes the site of genesis of action potentials that travel down myelinated axons via saltatory conduction (Leterrier 2016, 2018; Nelson and Jenkins 2017). Its precise functioning and intracellular localization contribute to maintain neuronal polarity, intrinsic neuronal excitability, thereby impacting local excitatory/inhibitory balance and hence, epileptogenesis (Wimmer et al. 2010; Vacher and Trimmer 2012; Leterrier 2016, 2018; Nelson and Jenkins 2017). The structure hubs a high concentration of voltage-gated K^+^ and Na^+^ channels that are mutated in adult epilepsy, cell-adhesion molecules (NF186 and NrCAM) and protein kinases (CK2, Cdk5, and CAMKII) among other proteins (Wimmer et al. 2010; Vacher and Trimmer 2012; Leterrier 2016, 2018; Nelson and Jenkins 2017). In this structure (see Fig. 4), the scaffolding protein Ankyrin-G anchors these channels and cell adhesion molecules via to α-β-spectrin tetramers that associate with the actin filaments (Leterrier 2016, 2018). Ankyrin-G binds kinesin to transport Na^+^ channels to the site (Barry et al. 2014) as well as to the MT-plus binding proteins EB1 and EB3 to link MTs in the AIS for axonal sorting (Leterrier et al. 2011). Conversely, Ankyrin’s association with Nde1l drives back in a dynein-dependent manner the entry of somato-dendritic cargoes into the axon (Kuijpers et al. 2016; Hamdan et al. 2020; Ye et al. 2020). By selecting these cargoes, the Ndel1/Ankyrin complex together with molecular motors, actin and MT cytoskeleton maintains neuronal polarity and axonal identity. Thus, alterations in the functions of Ndel1, cytoskeletal protein complexes and/or in molecular motors at the AIS could contribute to adult epileptogenesis by disturbing intrinsic neuronal excitability and polarity (Fig. 4). In summary, the AIS may be the physical point of convergence between channelopathies and cytoskeletal dysfunction in adult epilepsy.

**Figure 5:**
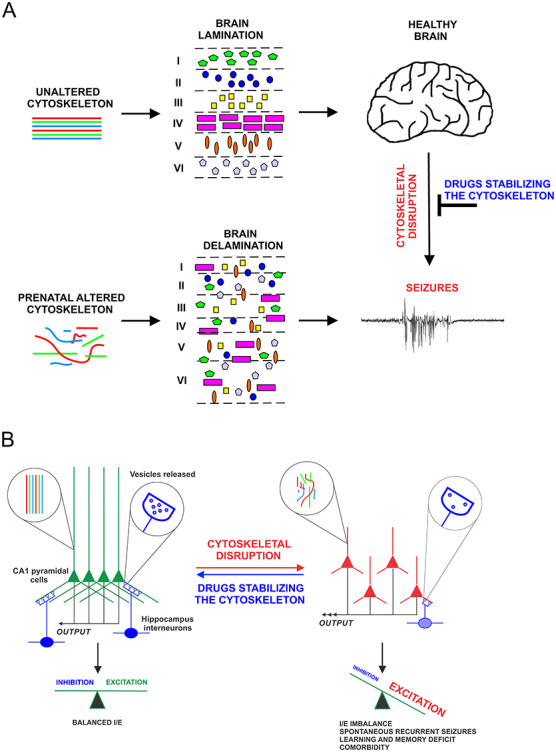
Working model on the postnatal roles of the cytoskeleton in adult epileptogenesis. (*A*) In developing neocortex, disruption of the cytoskeleton alters neuronal migration leading ultimately to misplacement of early born neurons, abnormal cortical lamination, and brain malformation. These developmental defects underlie pediatric epilepsy. In the adult brain that has formed properly, a collapse of the cytoskeleton can trigger changes in the structure → morphology → localization → functions of neurons that will ultimately lead to seizure activity. (*B*) These molecular, cellular, and anatomical deficits (i.e., ultrastructural collapse of the cytoskeleton, reduced inhibitory drives, loss of synapses, dispersion of neurons) cause an imbalance in the inhibitory/excitatory inputs, impede brain connectivity and even promote neuronal cell death. As a result, SRS may appear in specific brain regions. In the adult brain, cytoskeletal disruption may be triggered by acute or chronic pathological conditions such as trauma, stroke, ministrokes, neurodegenerative conditions, and perhaps by somatic de novo mutations in cytoskeletal proteins in subsets of adult neurons.

## Conclusion

The current review challenges the preconceived notion that postnatal alterations in the cytoskeleton are simply consequences or bystander effects of seizures. Recent studies from our lab and from other research groups, representing both human and nonhuman data, provide strong evidence that in some instances, cytoskeletal alterations can modify seizure activity, and can even cause seizures, and not simply the converse (see Fig. 5 for the working model). These findings may be relevant to numerous epileptic cases with no apparent prenatal causes such as brain malformations. The mechanisms causing cytoskeletal disruption in the adult brain are numerous, ranging from head trauma to stroke to neurodegenerative conditions, and remain to be further investigated in the context of epileptogenesis. While the Ndel1 CKO mouse affords us an opportunity to investigate novel mechanisms of epileptogenesis and related comorbidities, other experimental approaches are required to fully elucidate the role of the cytoskeleton in adult epileptogenesis. For example, the generation of tamoxifen-inducible Ndel1 KO mice to turn on and off Ndel1 expression in specific populations of hippocampal and/or cortical neurons in adulthood will allow us to understand in a spatio-temporal manner the involvement and plasticity of the cytoskeleton in epilepsy. Along this line, one can ask the question, is the AIS the primary intracellular site where this cytoskeleton-channel interface becomes dysfunctional in epilepsy? Finally, channelopathies are an established cause for adult epilepsy but very little is known about their contribution to brain malformation and pediatric epilepsy until recently. Indeed, a prenatal role for channelopathies is just being advanced to explain certain neurodevelopmental alterations and brain malformations (Smith and Walsh 2020). In contrast, cytoskeletal disruption can cause pediatric epilepsy, but in the adult, causality is still subject to debate. Thus, there are important parallels to be made between the mechanisms underlying pediatric epilepsy and those causing seizures in the adult. With the appropriate tools and approaches, future exciting discoveries revealing the postnatal role of the cytoskeleton in adult epileptogenesis are expected.

## Notes

We thank Dr Denise Ng at the University of Calgary for discussions on the surgically resected human tissues. *Conflict of Interest*: None declared.
